# Supplementary material for: A signal processing and deep learning framework for methylation detection using Oxford Nanopore sequencing
Source: Nat Commun. 2024 Feb 16;15:1448. doi: 10.1038/s41467-024-45778-y (PMC10873387; doi:10.1038/s41467-024-45778-y)
Supplement: Supplementary file 1 — Supplementary Information [file 41467_2024_45778_MOESM1_ESM.pdf]

Supplementary Information

A signal processing and deep learning framework for methylation detection using Oxford Nanopore sequencing

Mian Umair Ahsan[1], Anagha Gouru[1,2], Joe Chan[1], Wanding Zhou[3,4], Kai Wang[1,4*]

[1] Raymond G. Perelman Center for Cellular and Molecular Therapeutics, Children's Hospital of Philadelphia, Philadelphia, PA 19104, USA

[2] Department of Biology, University of Pennsylvania, Philadelphia, PA 19104, USA

[3] Center for Computational and Genomic Medicine, Children's Hospital of Philadelphia, Philadelphia, PA 19104, USA

[4] Department of Pathology and Laboratory Medicine, Perelman School of Medicine, University of Pennsylvania, Philadelphia, PA 19104, USA

[*] To whom correspondence should be addressed. Email: wangk@chop.edu

Supplementary Figures

Supplementary Figure 1. Per-site performance evaluation of DeepMod2 and other state of the art methylation callers on NIH3T3 genome evaluated against Illumina Mouse Methylation BeadChip array. a) shows heatmap and Pearson correlation coefficients as well as number of CpG sites used in the analysis (counting forward and reverse strands separately). b) shows precision, recall and F1-scores of DeepMod2 and other Nanopore methylation callers.

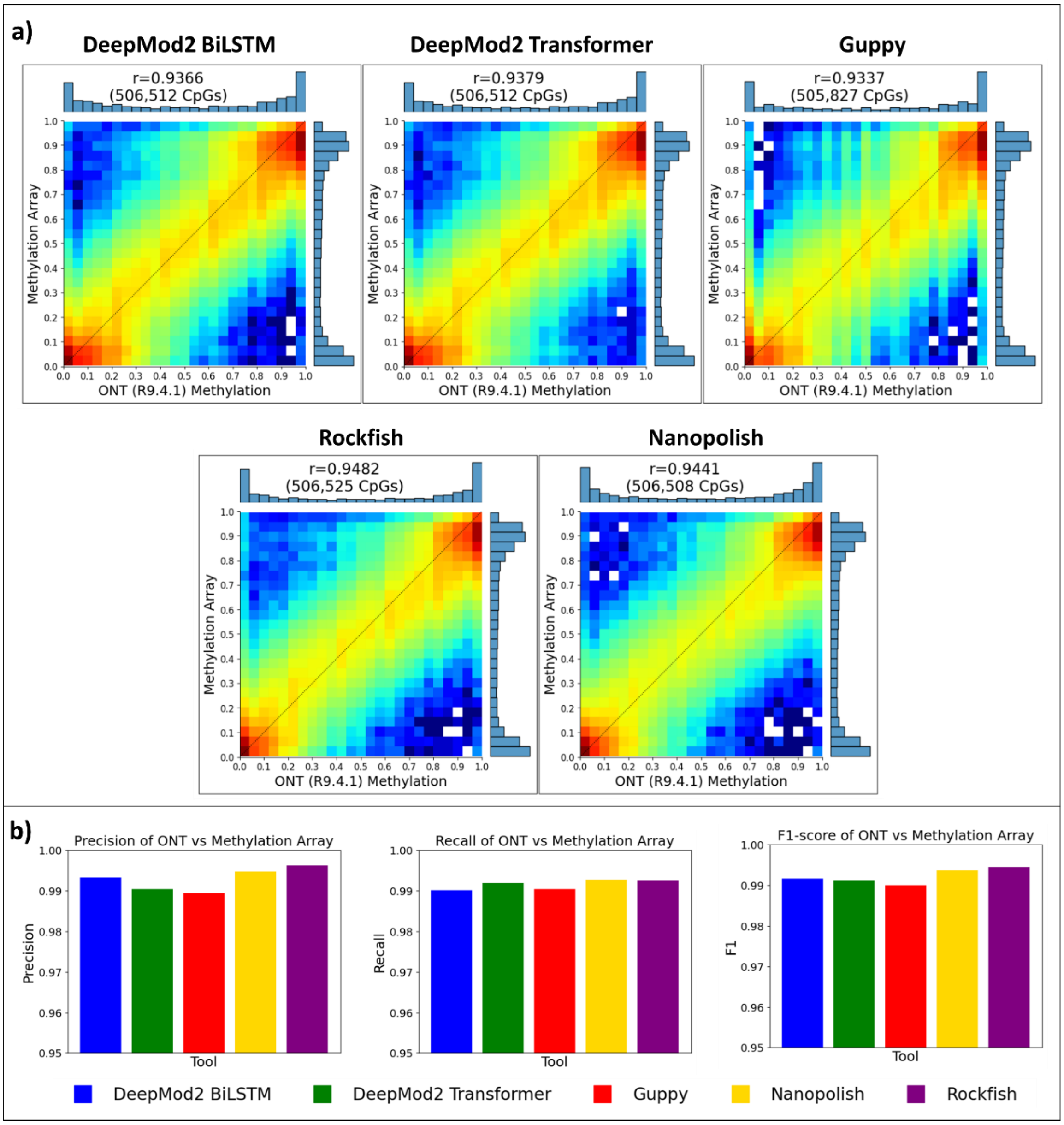

Supplementary Figure 2. Comparison of DeepMod2 and Guppy methylation calls in chr1 of HG002 R10.4.1 dataset. a) shows heatmap and Pearson's correlation of per-site methylation frequencies of DeepMod2 and Guppy. The panel on the left shows that there are several sites hypermethylated in Guppy compared to DeepMod2, i.e. DeepMod2 predicts methylation near 50% but Guppy predicts close to 100% methylation; these are shown in red circle. Whereas CpGs circles in black are those that DeepMod2 tends to predict as hypermethylated compared to Guppy. After counting SNVs as unmethylated in Guppy prediction, we see that the correlation between DeepMod2 and Guppy increases, as shown by the panel on the right, and the apparent over-methylation of Guppy is eliminated. b-c) show the heatmap and correlation between per-read probabilities from deep-learning models of Guppy and DeepMod2. b) shows correlation between all per-read prediction, whereas c) shows correlation for only those CpG sites that are hypermethylated in DeepMod2 and are shown in black circle in a).

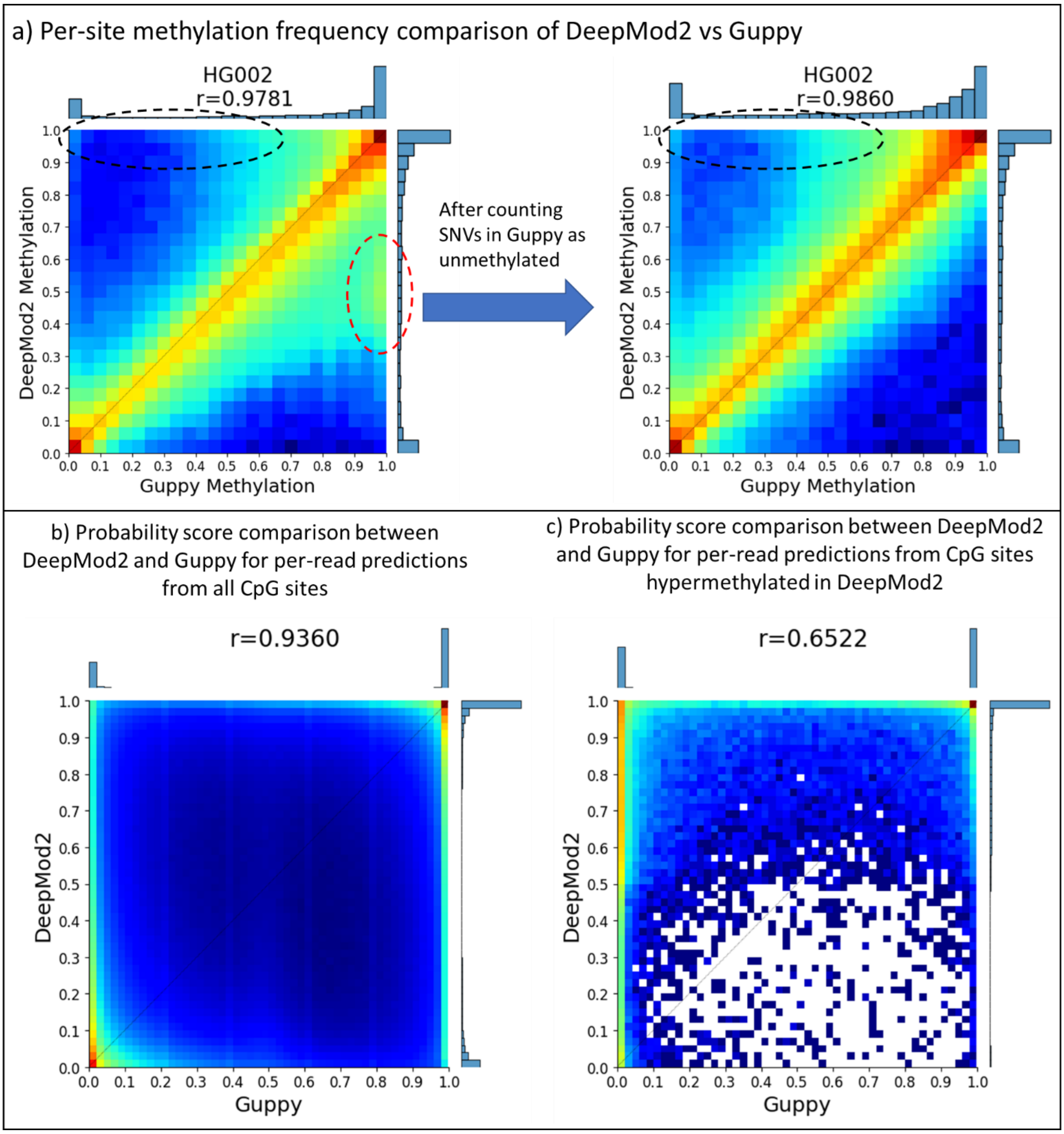

Supplementary Figure 3. ICR in chr20:37522341-37522993 (GRCh38) that was not detected in ONT datasets of HG002, HG003 and HG004. The IGV plots show that both haplotypes of each genome are almost completely methylated, thus showing no differential methylation. The reads of each genome are grouped by phase, and methylated and unmethylated cytosines are colored red and blue, respectively. This figure is generated in IGV.

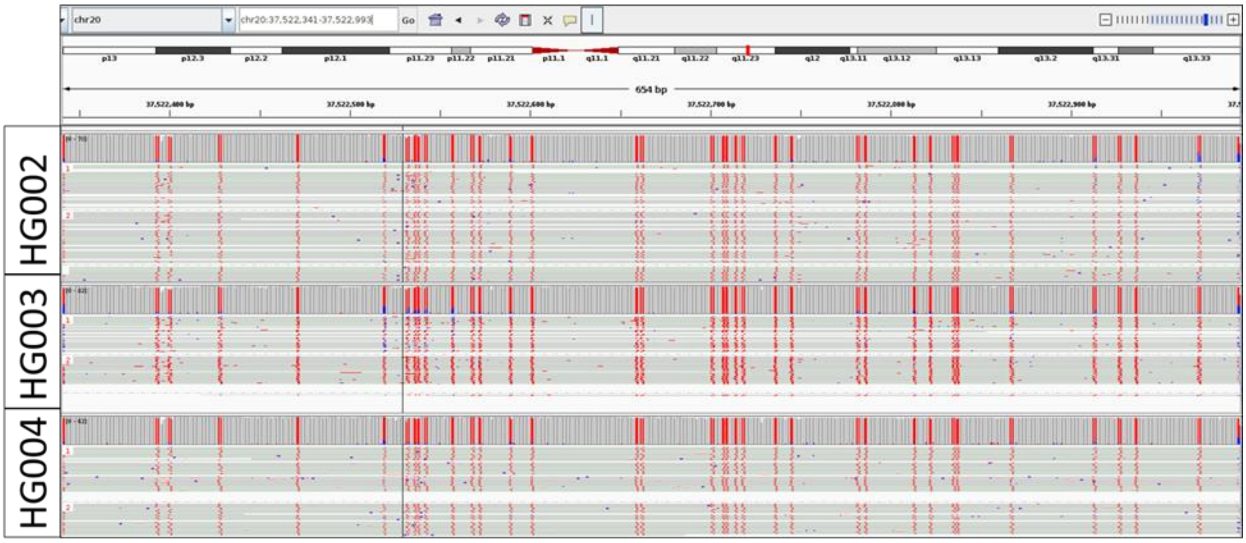

Supplementary Figure 4. ICR in chr20:37520202-37521842 (GRCh38) that was detected in ONT datasets of HG002 and HG003, but not in HG004. The IGV plot of HG004 shows that both haplotypes are almost completely methylated apart from 5 CpG sites, thus showing no differential methylation. The reads of each genome are grouped by phase, and methylated and unmethylated cytosines are colored red and blue, respectively. This figure is generated in IGV.

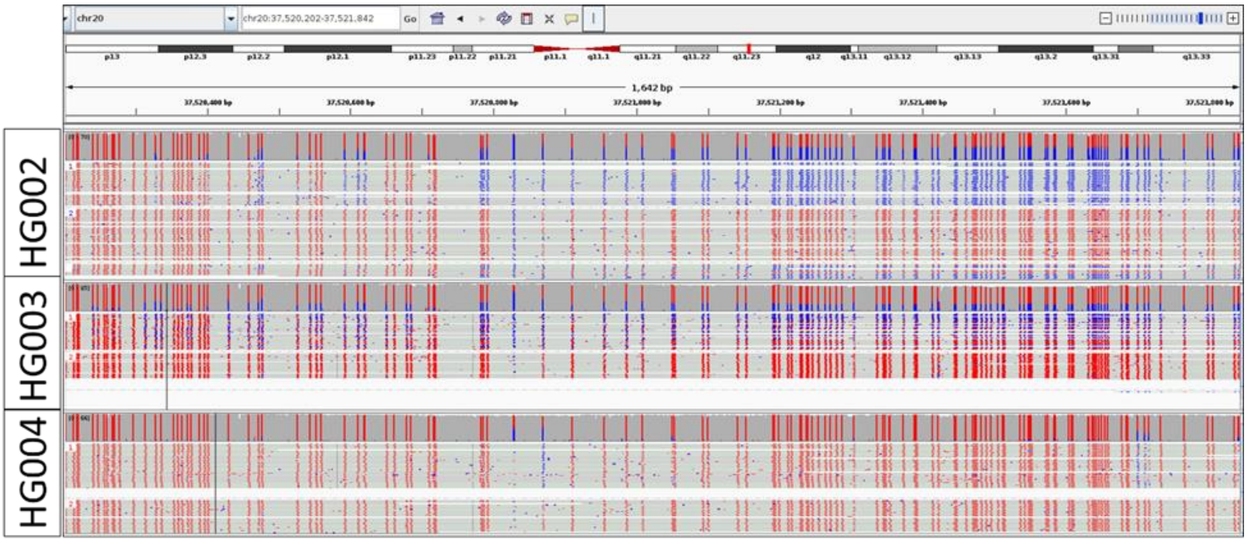

Supplementary Figure 5. ICR in chr20:43513725-43515256 (GRCh38) that was detected in ONT datasets of HG003 and HG004, but not in HG002. The IGV plot of HG002 shows that although the region is semi-methylated, all the reads are unphased and thus not haplotype specific methylation was available. This region lies within a long stretch of homozygosity due to which no phasing could be performed. The reads of each genome are grouped by phase, and methylated and unmethylated cytosines are colored red and blue, respectively. This figure is generated in IGV.

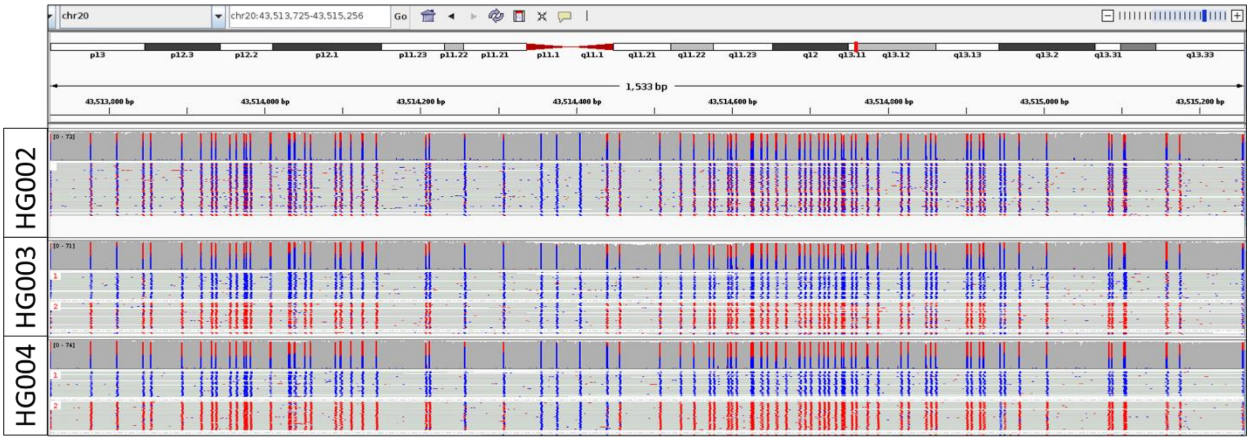

Supplementary Figure 6. IGV plot of methylation in HG002 genome inside a 5,218bp long insertion located at chr1:103,938,232 (GRCh38). DeepMod2 detects methylation for all CpG loci on a reads, even if they are not mapped to any reference coordinates, such as the bases within the insertion shown here. DeepMod2 adds methylation tags to BAM file for all CpG loci on a read, and the read can be mapped later to a different reference sequence. The top panel shows methylation from alignment to GRCh38 and the bottom panel shows alignment of the same reads to the inserted sequence as reference. After aligning to the inserted sequence, we can examine methylation patterns inside the insertion, without needing to perform methylation calling from scratch. Methylated and unmethylated cytosines are colored red and blue, respectively. This figure is generated in IGV.

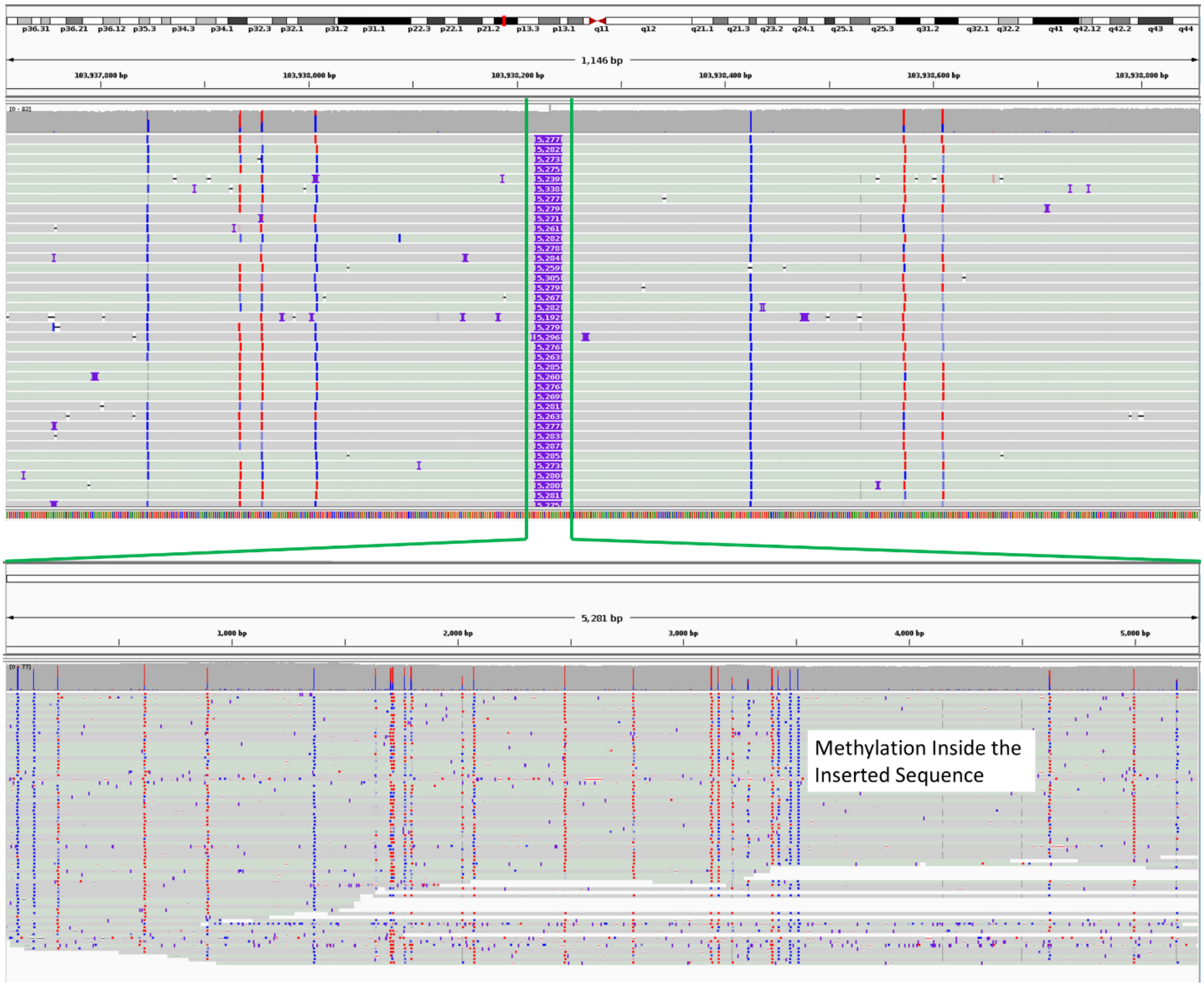

Supplementary Figure 7. IGV plot of methylation in HG002 genome inside a heterozygous 1,198bp long inversion located at chr1: 197,787,659 (GRCh38). The upper two panels show ONT reads aligned to GRCh38 with top panel showing the view of SNVs and the second panel showing view of methylation. The primary and supplementary alignments in these panels are linked together but colored differently, which illustrates the inversion. However, methylation within and near the inversion is not visible due to supplementary alignments. After realigning these reads to the inverted sequence, all the reads have primary alignments and methylation within the inversion becomes visible, as shown in the bottom two panels. Methylated and unmethylated cytosines are colored red and blue, respectively. This figure is generated in IGV.

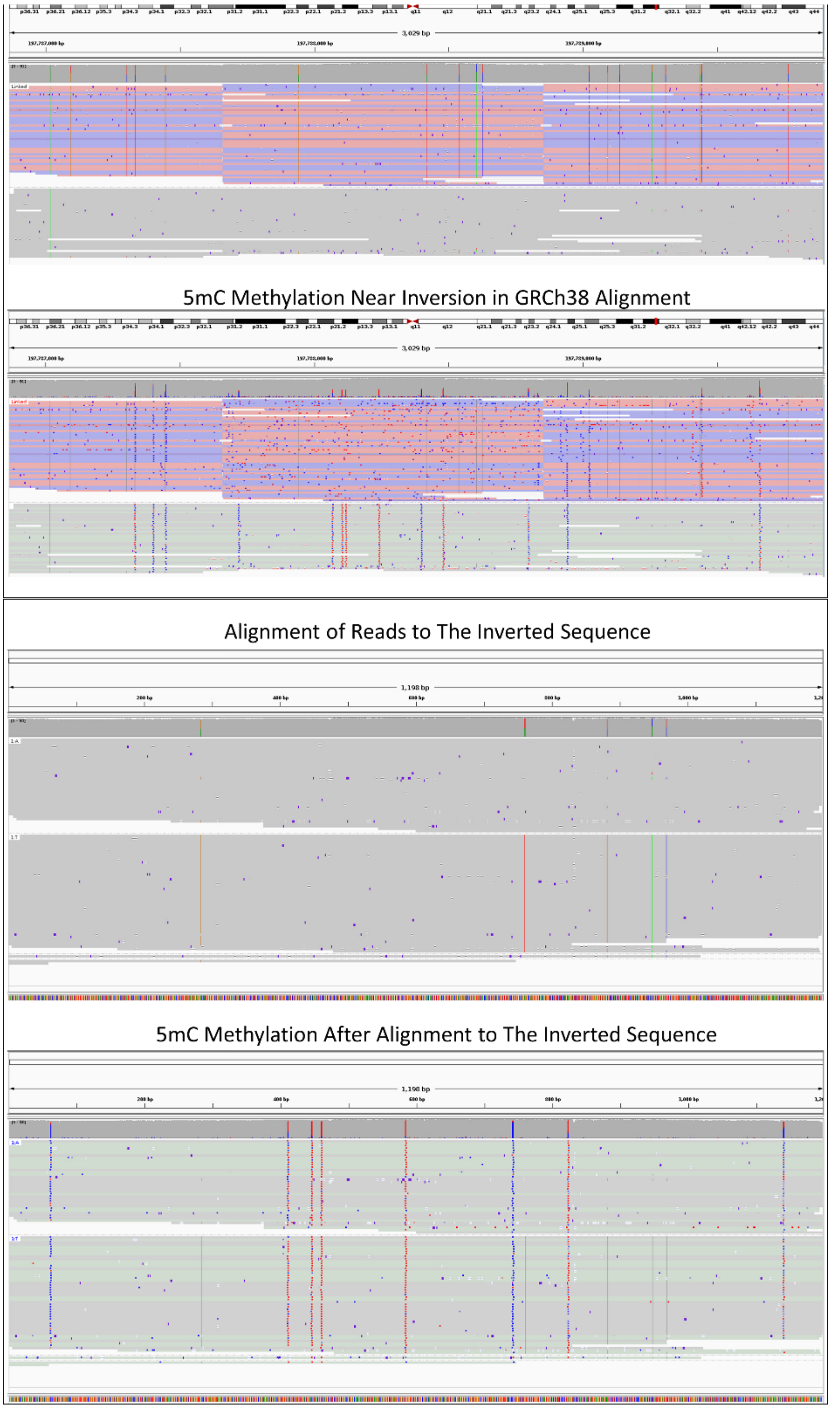

Supplementary Figure 8. Details of DeepMod2 model pruning for BiLSTM and Transformer models.

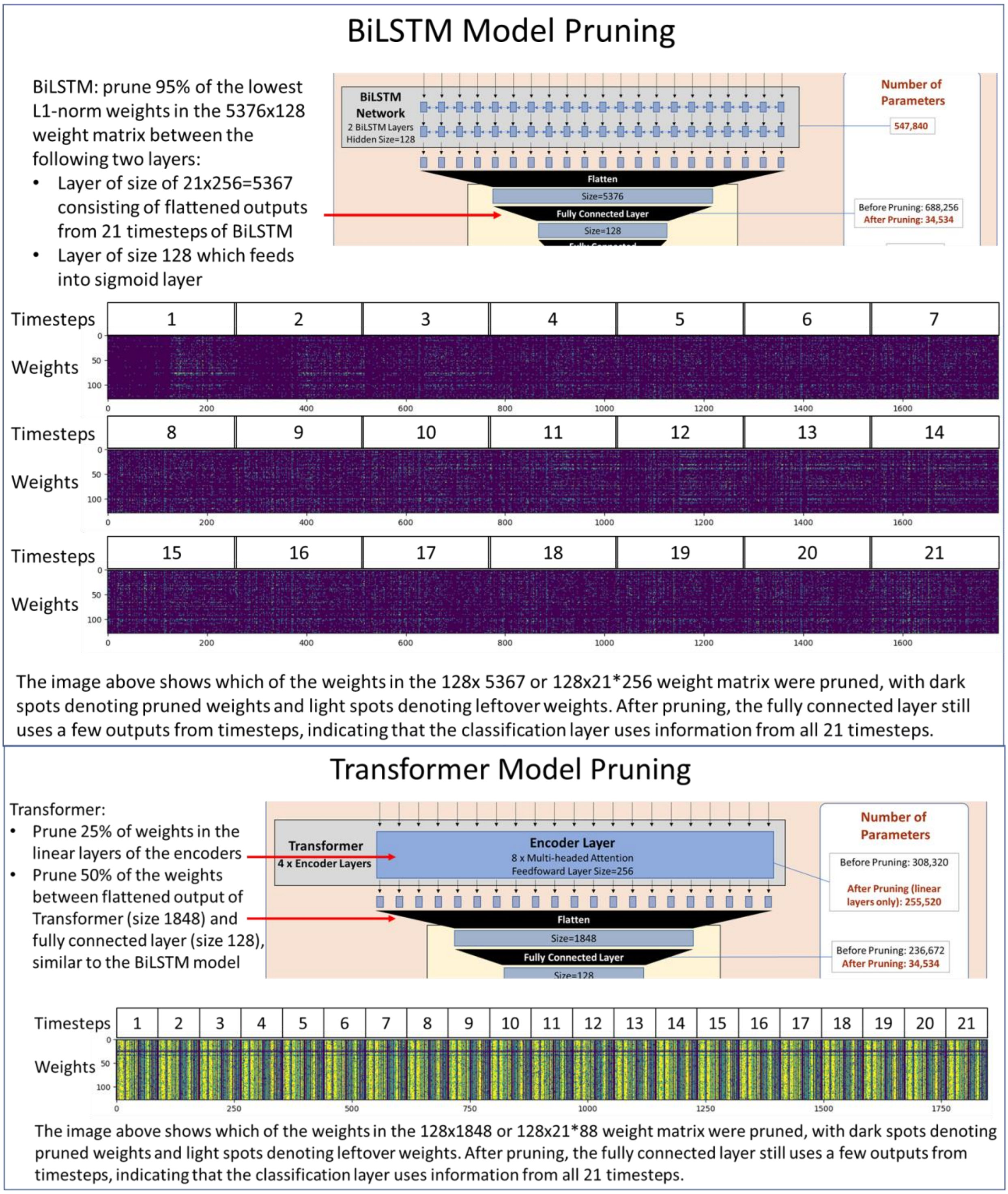

Supplementary Figure 9. Nanopore read signals aligned to basecalled read sequences using move tables in a 21bp window centered at chr1:517959 (GRCh38) of HG002 genome. The left panel shows signals from three reads sequenced using R9.4.1 flowcells, whereas the panel on the right shows signal from three reads sequenced using R10.4.1 flowcells.

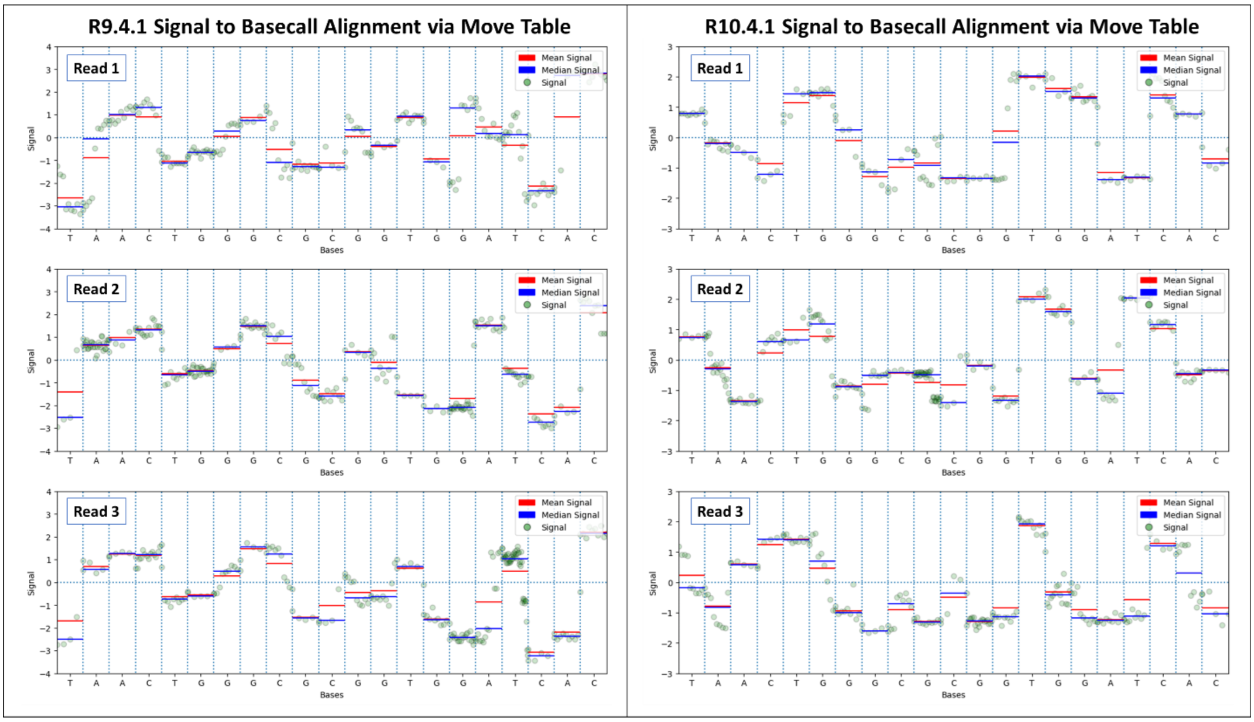

Supplementary Figure 10. Details of DeepMod2 Transformer model.

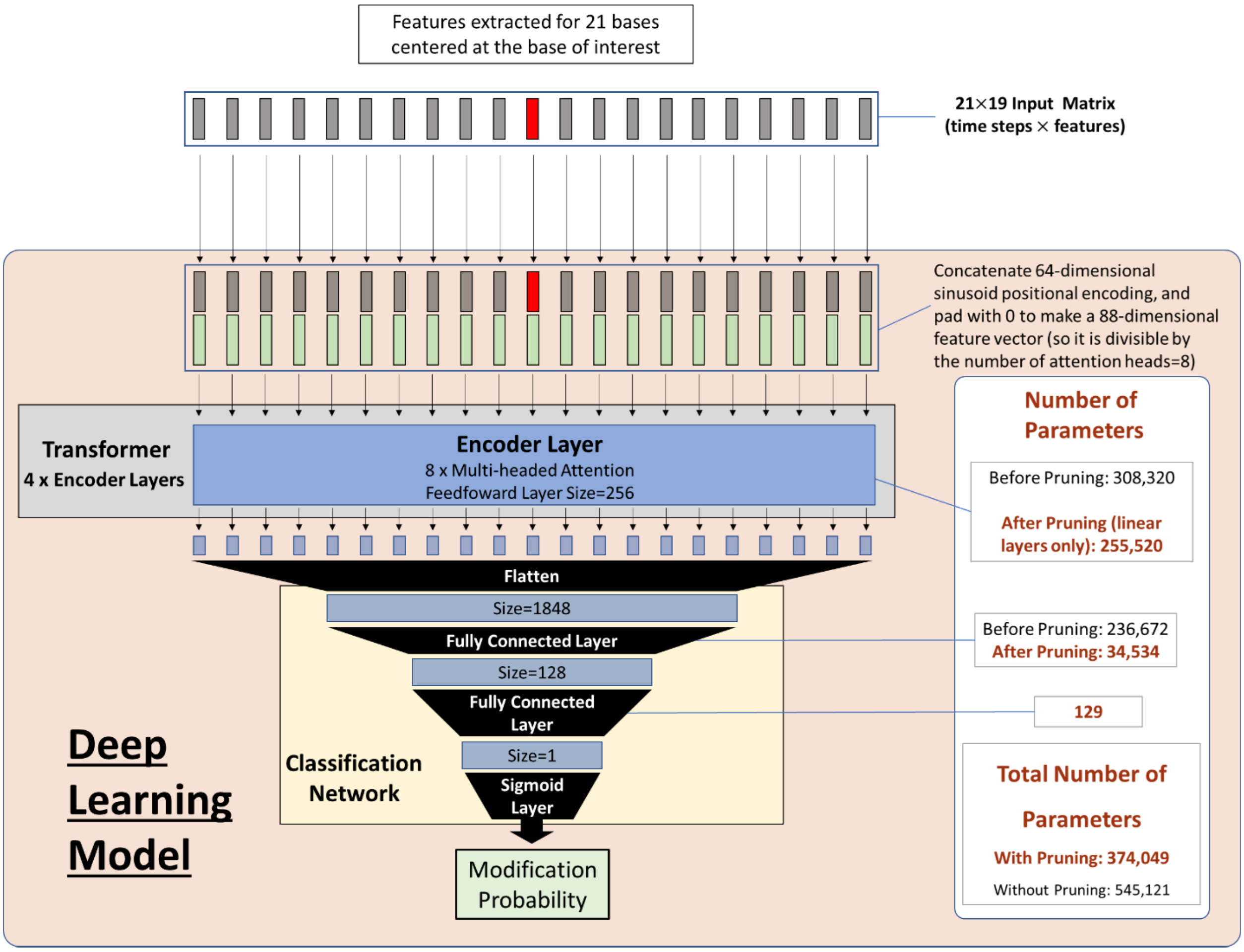

Supplementary Tables

Supplementary Table 1. Runtime performance of DeepMod2 and Dorado, measured in hours, on a single PromethION flowcell dataset of HG004 using 16 Intel Xeon Gold 5317 3GHz CPUs and 1 NVIDIA A100 GPU. The comparison is shown using three basecaller models, FAST, HAC and SUP, and steps that used GPU are marked with *.

**DeepMod2 Methylation Calling Framework Runtime**							
**Dorado Basecaller Model**	**FAST**		**HAC**			**SUP**	
**DeepMod2 Device Configuration**	**CPU**	**GPU**	**CPU (Without Model Pruning)**	**CPU**	**GPU**	**CPU**	**GPU**
Dorado Basecalling	5.5*	5.5*	5.5*	5.5*	5.5*	26.2*	26.2*
Minimap2 Alignment	5.4	5.4	7.2	7.2	7.2	7.5	7.5
**DeepMod2 Methylation Calling**	**13.8**	**5.8***	**60.1**	**13.2**	**5.7***	**13.1**	**5.7***
Total	24.7	16.7	72.8	25.9	18.4	46.8	39.4

**Dorado Methylation Calling Framework Runtime**			
**Dorado Basecaller Model**	**FAST**	**HAC**	**SUP**
Dorado Basecalling and Methylation Calling	2.7*	6.1*	26.9*
Minimap2 Alignment and SAMtools Sorting Indexing	7.1	9.6	9.8
Modkit Pileup	0.5	0.5	0.5
Total	10.3	16.1	37.1

Supplementary Table 2. Comparison of algorithm and models of orignal DeepMod and DeepMod2.

DeepMod2	Original DeepMod
Contains models for R10.4.1 and R9.4.1 flowcells	Contains models for R9.4.1 flowcells
Compatible with Guppy and Dorado Basecallers	Compatible only with deprecated Metrichore and Albacore v1 Basecallers
Uses move tables for signal alignment	Uses event tables with predicted k-mers for signal alignment
Requires BAM file to be aligned externally and provided as input.	Aligns reads to reference genome internally via BWA-MEM or minimap2 to align signal to reference genome
Can call methylation from unaligned reads, unmapped or clipped segments of an aligned read.	
Calls methylation on reference CpG loci and read CpG loci	Calls methylation on reference CpG loci only
Produces BAM output with methylation tags added	Discards any alignments generated during runtime and saves per-read prediction in HDF5 format
BiLSTM model uses 19 features per base	BiLSTM model uses 7 features per base
BiLSTM model has 2 layers of size 128	BiLSTM model has 3 layers of size 100
BiLSTM models uses output from all timesteps for per-read methylation prediction.	BiLSTM models uses output only from middle timestep for per-read methylation prediction
Does not use any such network	Applies an additional neural network on per-site predictions to update methylation levels based on methylation levels of nearby CpG sites
Trained on native methylation data from chr1-21 of HG002, HG003 and HG004, with short-read sequencing ground truth labels	Trained on synthetically modified and unmodified E. coli datasets
Provides phased methylation calls	

Supplementary Table 3. Per-site performance of original DeepMod and DeepMod2 on chr21 of HG001 R9.4.1 dataset.

Region	Tool	TP	FP	FN	TN	Precision	Recall	F1	Correlation
**Genomewide**	**DeepMod**	65894	507	18204	59197	0.9924	0.7835	0.8757	0.7242
	**DeepMod Plus Clustering**	81776	1368	2322	58336	0.9835	0.9724	0.9779	0.8555
	**DeepMod2**	83592	1030	506	58674	0.9878	0.9940	0.9909	0.9133
**CPG_Island**	**DeepMod**	4273	58	729	9584	0.9866	0.8543	0.9157	-
	**DeepMod Plus Clustering**	4946	81	56	9561	0.9839	0.9888	0.9863	-
	**DeepMod2**	4981	43	21	9599	0.9914	0.9958	0.9936	-
**CPG_Shelves**	**DeepMod**	7063	30	1993	3419	0.9958	0.7799	0.8747	-
	**DeepMod Plus Clustering**	8789	109	267	3340	0.9878	0.9705	0.9791	-
	**DeepMod2**	8991	73	65	3376	0.9919	0.9928	0.9924	-
**CPG_Shores**	**DeepMod**	9968	68	2917	6812	0.9932	0.7736	0.8698	-
	**DeepMod Plus Clustering**	12473	168	412	6712	0.9867	0.9680	0.9773	-
	**DeepMod2**	12789	137	96	6743	0.9894	0.9925	0.9910	-
**Promoter**	**DeepMod**	2049	34	506	6241	0.9837	0.8020	0.8836	-
	**DeepMod Plus Clustering**	2500	74	55	6201	0.9713	0.9785	0.9748	-
	**DeepMod2**	2544	70	11	6205	0.9732	0.9957	0.9843	-
**Intron**	**DeepMod**	42870	226	12016	29402	0.9948	0.7811	0.8751	-
	**DeepMod Plus Clustering**	53320	705	1566	28923	0.9870	0.9715	0.9791	-
	**DeepMod2**	54532	490	354	29138	0.9911	0.9936	0.9923	-
**Exon**	**DeepMod**	9936	61	2535	8438	0.9939	0.7967	0.8845	-
	**DeepMod Plus Clustering**	12200	116	271	8383	0.9906	0.9783	0.9844	-
	**DeepMod2**	12407	79	64	8420	0.9937	0.9949	0.9943	-
**Intergenic**	**DeepMod**	12213	209	3437	19246	0.9832	0.7804	0.8701	-
	**DeepMod Plus Clustering**	15185	522	465	18933	0.9668	0.9703	0.9685	-
	**DeepMod2**	15565	429	85	19026	0.9732	0.9946	0.9838	-

Supplementary Table 4. Number of positive and negative labels or CpG sites per genome in both training and validation datasets.

**Number of CpG Sites In Training Dataset**				
	HG002 (chr2-21)	HG003 (chr2-21)	HG004 (chr2-21)	Total
Methylated	5,332,746	3,015,338	3,327,842	11,675,926
Unmethylated	2,490,174	2,725,528	2,075,748	7,291,450
Total	7,822,920	5,740,866	5,403,590	18,967,376

**Number of CpG Sites In Validation Dataset**				
	HG002 (chr22)	HG003 (chr22)	HG004 (chr22)	Total
Methylated	179,954	100,680	115,318	395,952
Unmethylated	65,730	63,116	54,388	183,234
Total	245,684	163,796	169,706	579,186

Supplementary Table 5. Number of positive and negative labels per genome in testing datasets, i.e. number of CpG sites in per-read evaluation.

**Number of CpG Sites In Per-Read Evaluation**				
	HG002 (chr1)	HG003 (chr1)	HG004 (chr1)	NIH3T3 (chr1-19)
Methylated	548,110	318,232	346,438	99,316
Unmethylated	269,195	272,461	222,531	52,250
Total	817,305	590,693	568,969	151,566

Supplementary Table 6. Number of CpG sites in per-site and correlation evaluation of each genome.

**Number of CpG Sites In Per-Site Evaluation**				
	HG002 (chr1)	HG003 (chr1)	HG004 (chr1)	NIH3T3 (chr1-19)
Methylated	1,087,680	731,932	746,534	6,190,882
Unmethylated	350,481	369,277	295,683	3,107,902
Total	1,438,161	1,101,209	1,042,217	9,298,784
**Number of CpG Sites In Correlation Analysis**				
	HG002 (chr1)	HG003 (chr1)	HG004 (chr1)	NIH3T3 (chr1-19)
Total	4,549,338	4,557,266	4,540,918	15,427,244
