# Supplementary material for: A signal processing and deep learning framework for methylation detection using Oxford Nanopore sequencing
Source: Nat Commun. 2024 Feb 16;15:1448. doi: 10.1038/s41467-024-45778-y (PMC10873387; doi:10.1038/s41467-024-45778-y)
Supplement: Supplementary file 3 — Description of Additional Supplementary Files [file 41467_2024_45778_MOESM3_ESM.pdf]

File Name: Supplementary Data 1-5
Description: Excel file containing Supplementary Data 1 to 5.
Supplementary Data 1: R9.4.1 and R10.4.1 flowcell per-read performance evaluation of DeepMod2 and other Nanopore methylation callers.
Supplementary Data 2: R9.4.1 flowcell per-site performance evaluation of DeepMod2 and other Nanopore methylation callers.
Supplementary Data 3: R10.4.1 flowcell per-site performance evaluation of DeepMod2 and other Nanopore methylation callers.
Supplementary Data 4: R9.4.1 and R10.4.1 flowcell per-site performance of DeepMod2 models with and without pruning.
Supplementary Data 5: per-site performance evaluation of Dorado versus DeepMod2 with or without reference alignment and features under different basecaller models.

File Name: Supplementary Data 6
Description: IGV plots of ONT RRMS methylation and coverage track figures for each chromosome.

File Name: Supplementary Data 7
Description: Putative imprinted regions in HG002, HG003 and HG004 detected by DSS using phased methylation calls from DeepMod2.

File Name: Supplementary Data 8
Description: Methylation microarray beta values of the NIH3T3 cell line.
