# Supplementary material for: A signal processing and deep learning framework for methylation detection using Oxford Nanopore sequencing
Source: Nat Commun. 2024 Feb 16;15:1448. doi: 10.1038/s41467-024-45778-y (PMC10873387; doi:10.1038/s41467-024-45778-y)

Supplementary Data 6

IGV plots of ONT RRMS methylation and coverage track figures for each chromosome

Mian Umair Ahsan[1], Anagha Gouru[1,2], Joe Chan[1], Wanding Zhou[3,4], Kai Wang[1,4*]

[1] Raymond G. Perelman Center for Cellular and Molecular Therapeutics, Children's Hospital of Philadelphia, Philadelphia, PA 19104, USA

[2] Department of Biology, University of Pennsylvania, Philadelphia, PA 19104, USA

[3] Center for Computational and Genomic Medicine, Children's Hospital of Philadelphia, Philadelphia, PA 19104, USA

[4] Department of Pathology and Laboratory Medicine, Perelman School of Medicine, University of Pennsylvania, Philadelphia, PA 19104, USA

[*] To whom correspondence should be addressed. Email: wangk@chop.edu

Each figure displays methylation levels of CpGs in blue track for HG002 ONT WGS and HG002 ONT RRMS, with corresponding coverage of each CpG site in green tracks, as viewed in IGV. "coverage" and

“mod_percentage” columns from aggregated per-site output of DeepMod2 were converted into BigWig format. In order to show a global view of BigWig tracks, IGV applies a mean function in a sliding window to simplify the view. We visualized the methylation and coverage for all CpG sites (shown by first four tracks in each figure) which shows enrichment of on-target regions compared to off-target regions as shown by coincidence of coverage peaks and RRMS track at the bottom. We separately visualized methylation and coverage for CpG sites only in RRMS regions, shown by next four tracks with “RRMS CpGs Only” in the description. We used bedtools intersect to select coverage and methylation from “All CpGs” tracks for only those CpGs that fell within on-target region used in RRMS. Comparison of methylation from RRMS and WGS for “RRMS CpGs” shows high correlation of methylation.

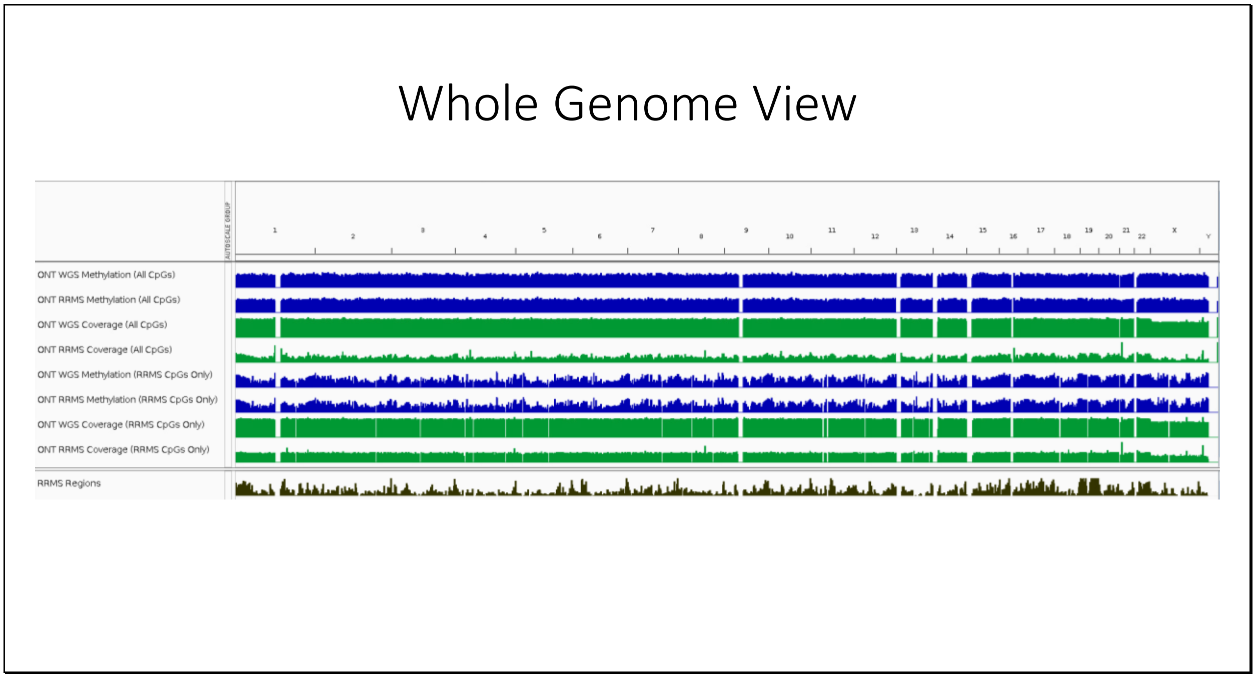

Chromosome 1

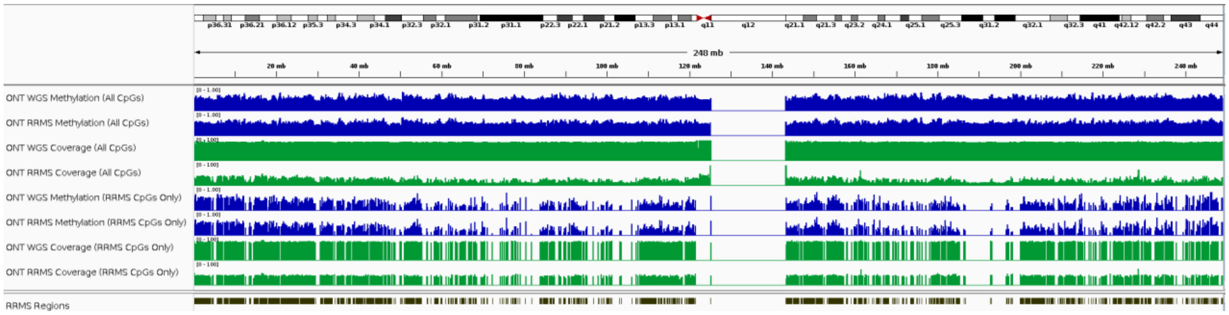

Chromosome 2

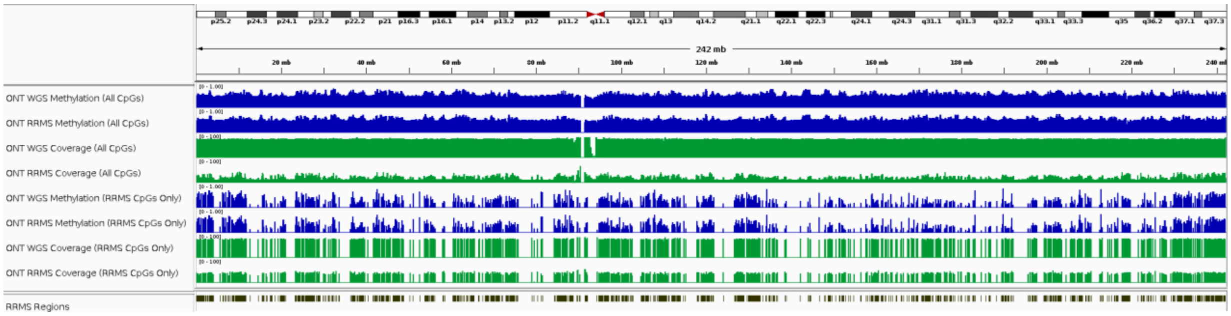

Chromosome 3

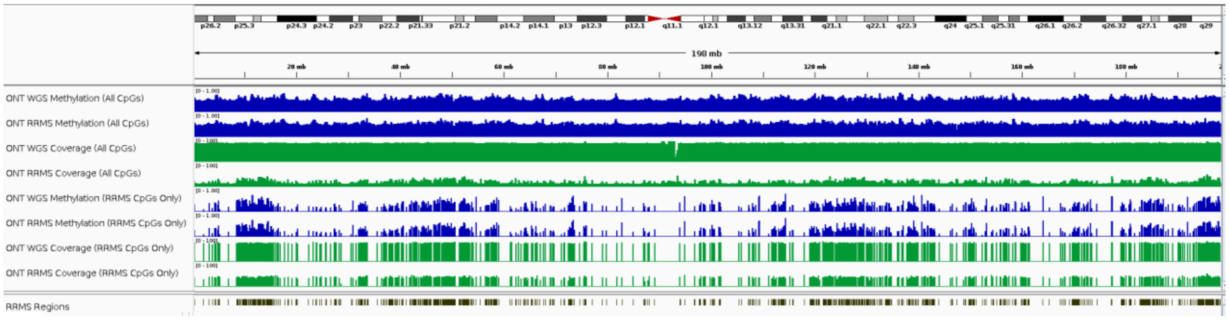

Chromosome 4

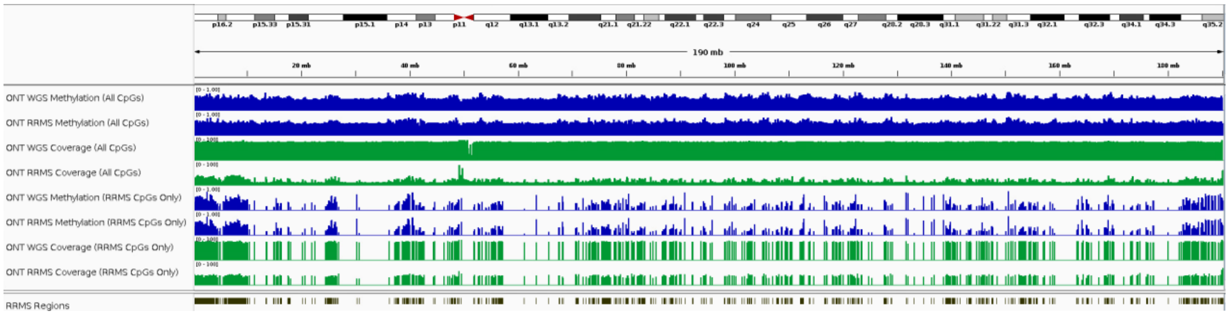

Chromosome 5

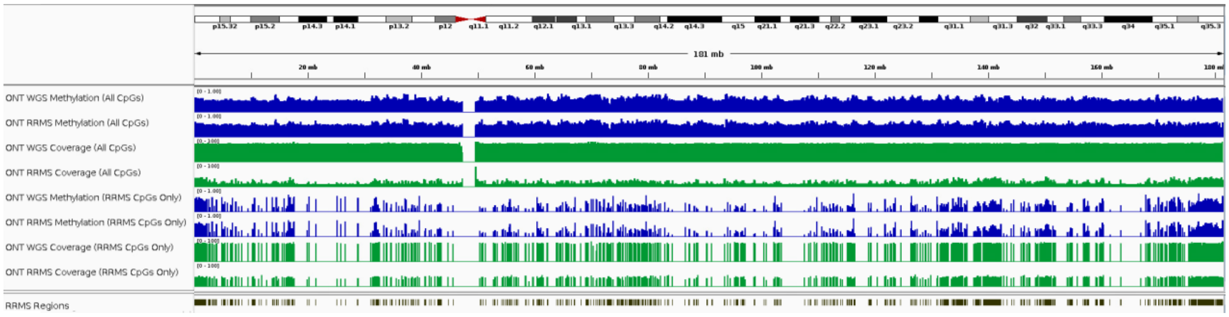

Chromosome 6

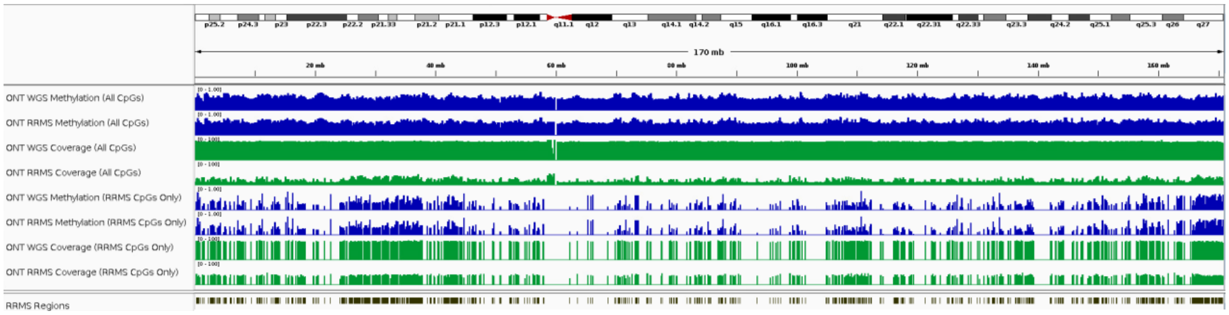

Chromosome 7

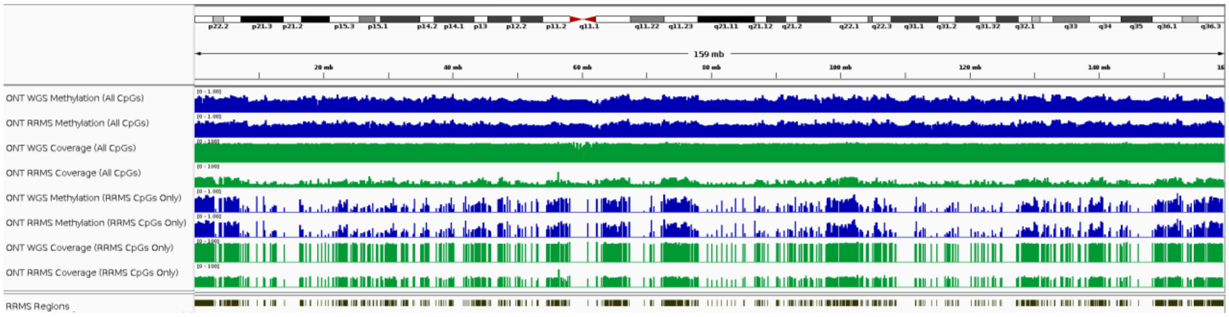

Chromosome 8

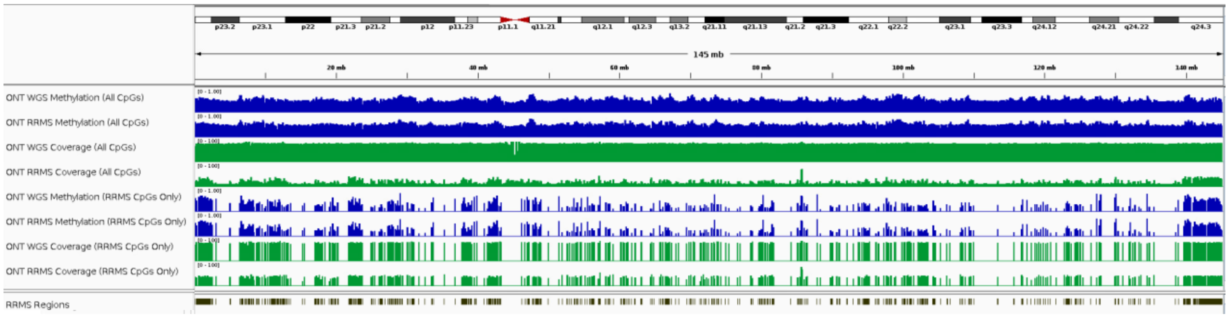

Chromosome 9

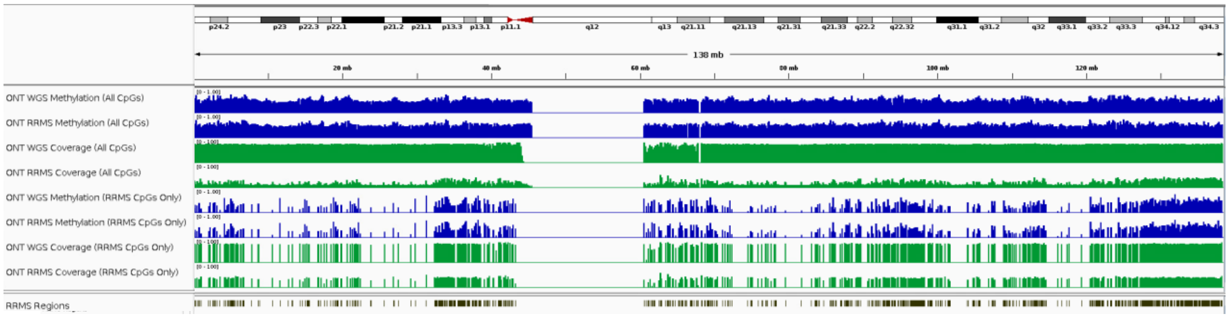

Chromosome 10

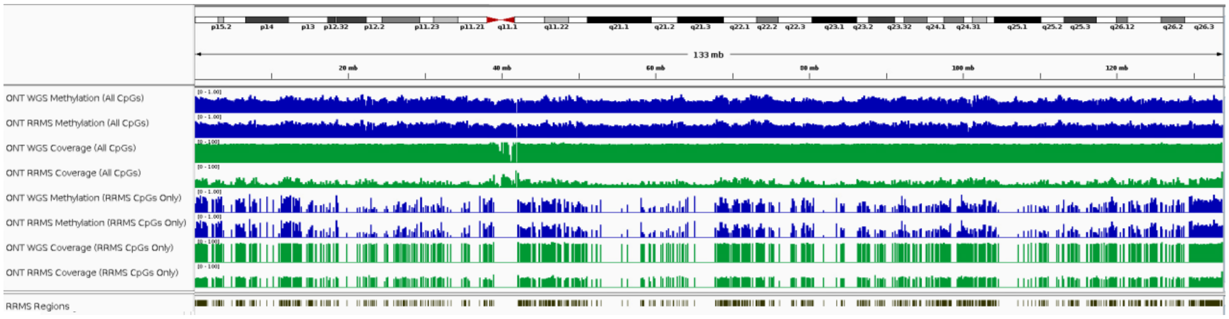

Chromosome 11

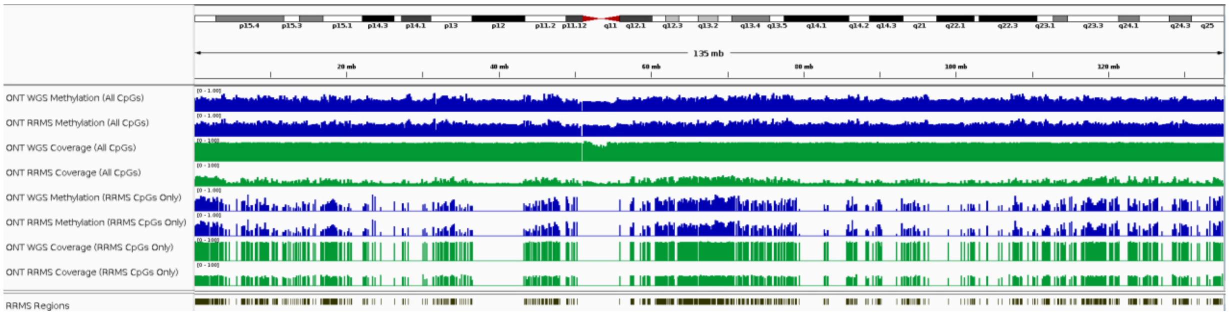

Chromosome 12

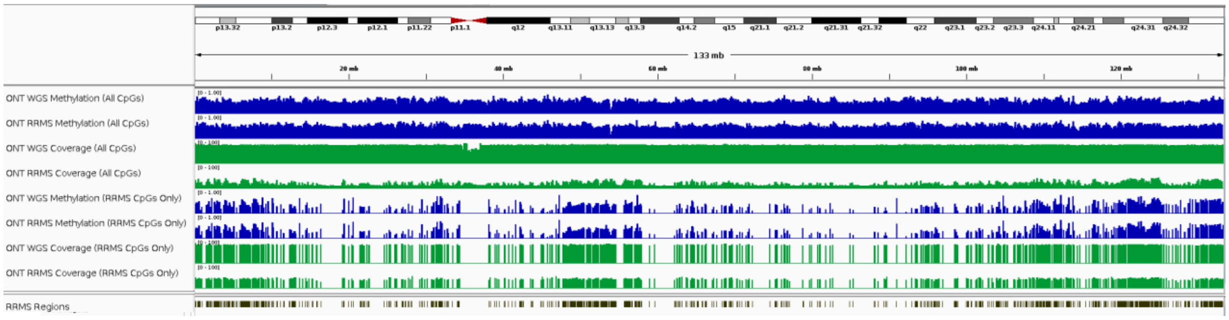

Chromosome 13

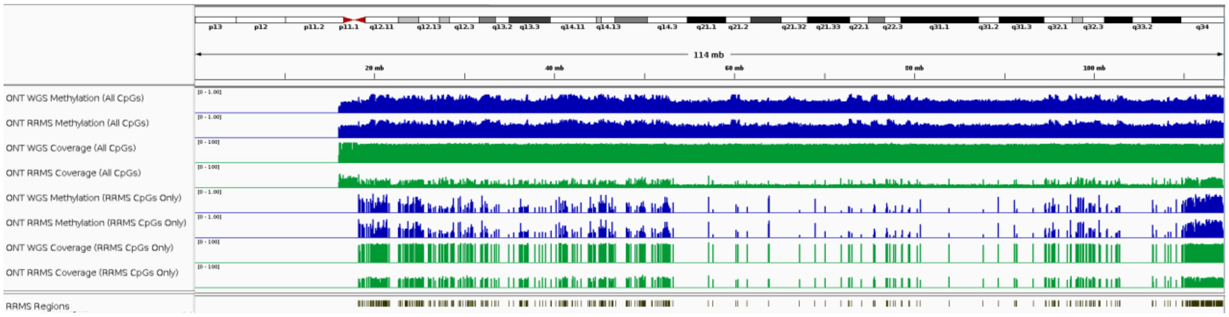

Chromosome 14

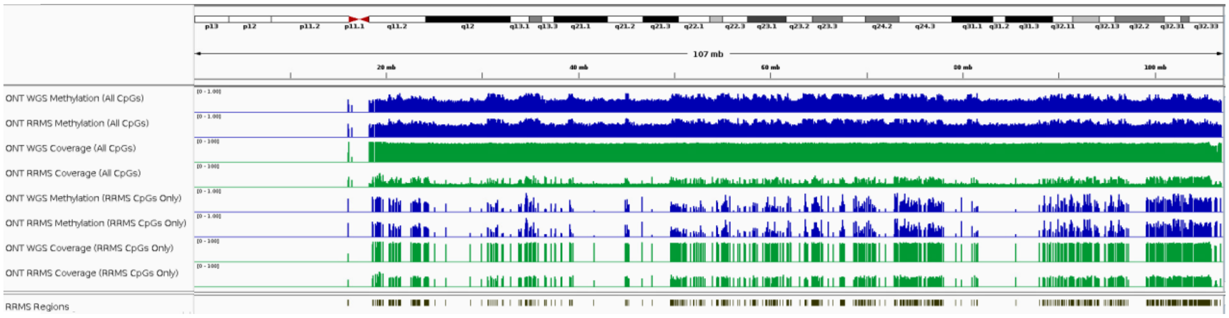

Chromosome 15

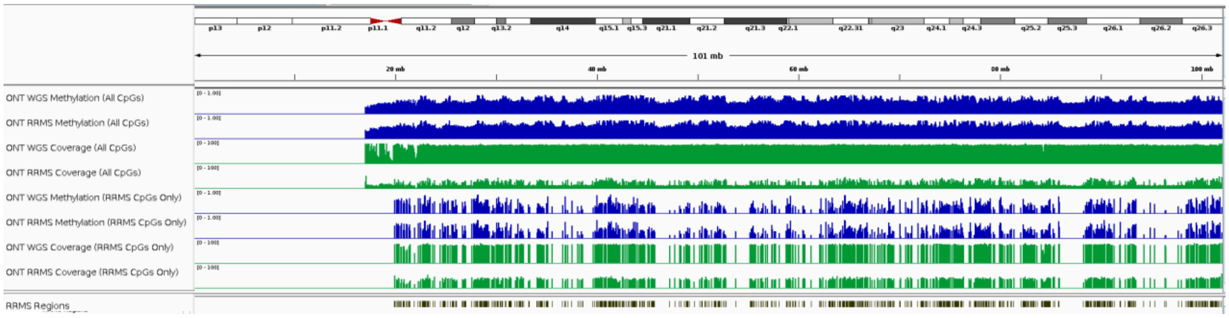

Chromosome 16

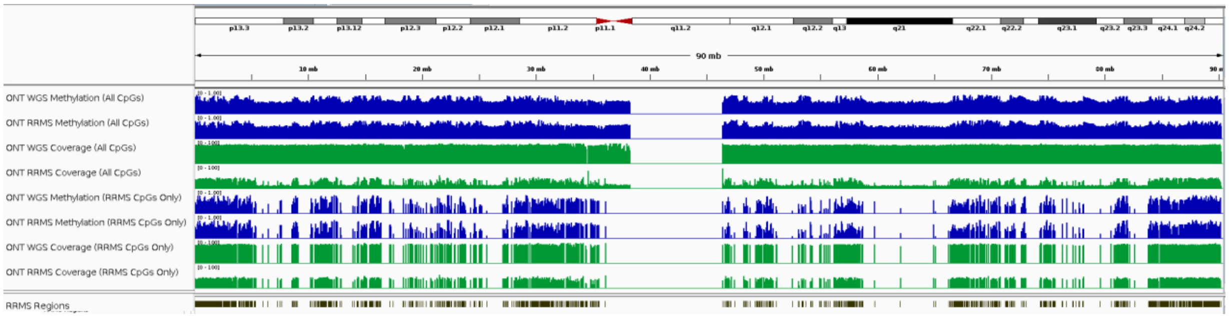

Chromosome 17

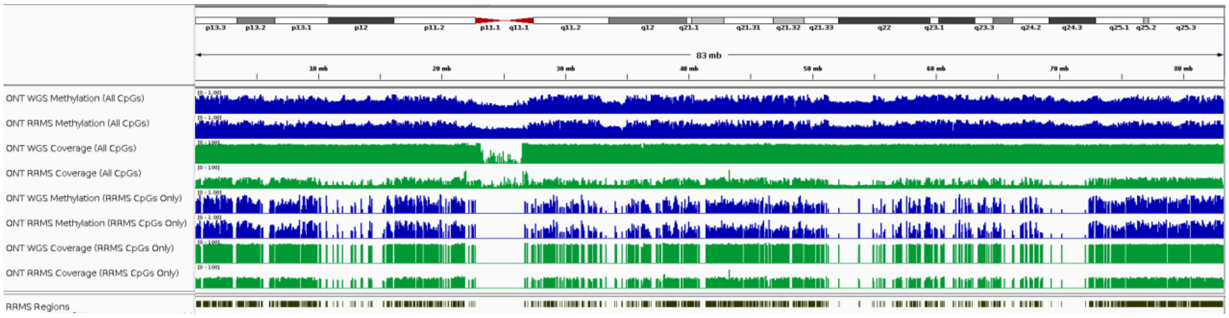

Chromosome 18

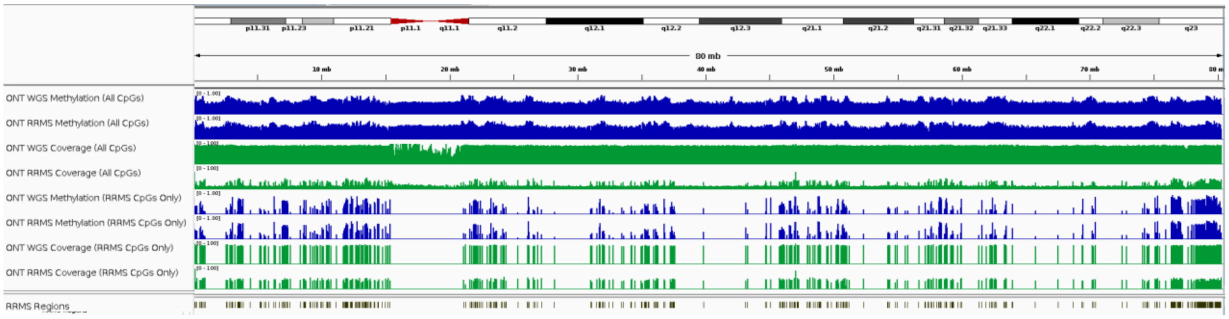

Chromosome 19

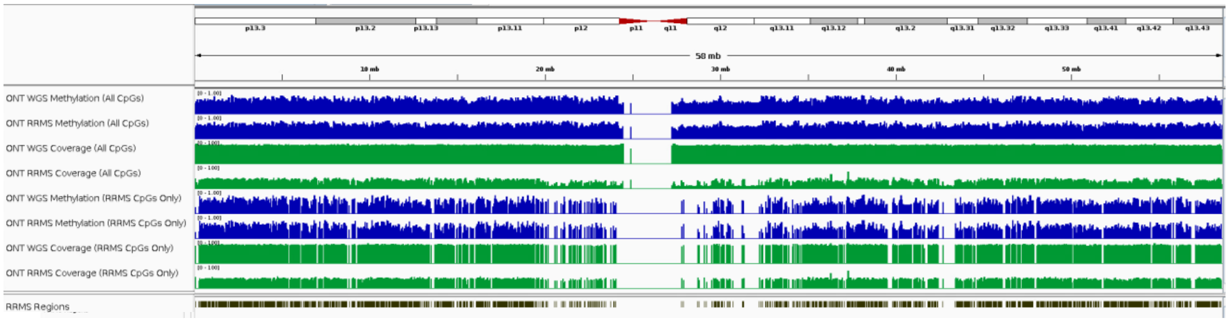

Chromosome 20

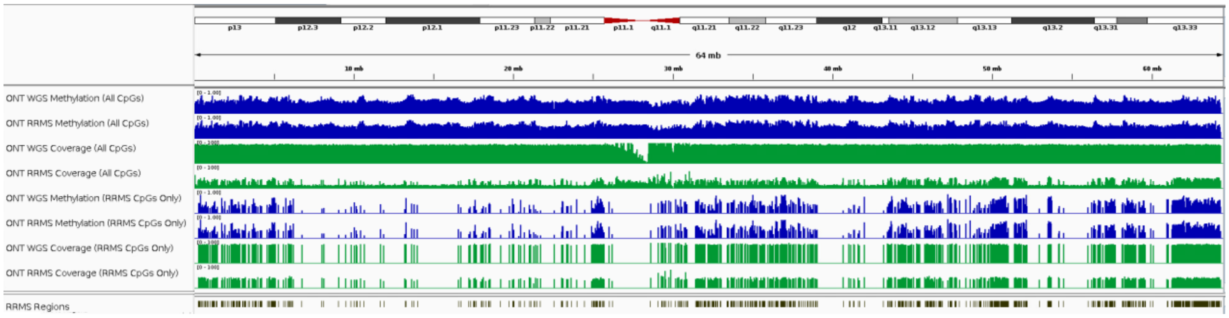

Chromosome 21

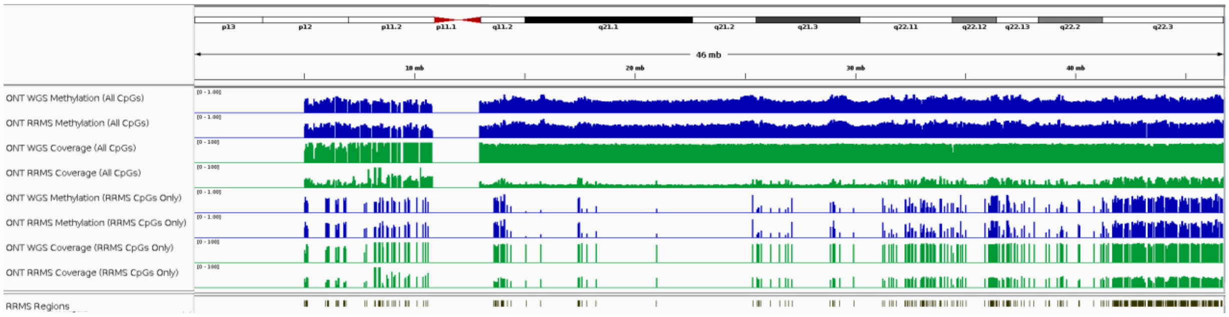

Chromosome 22

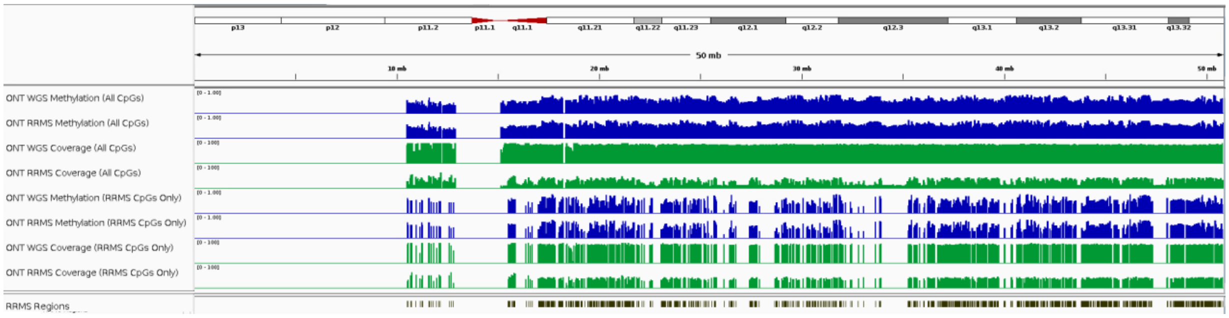

Chromosome X

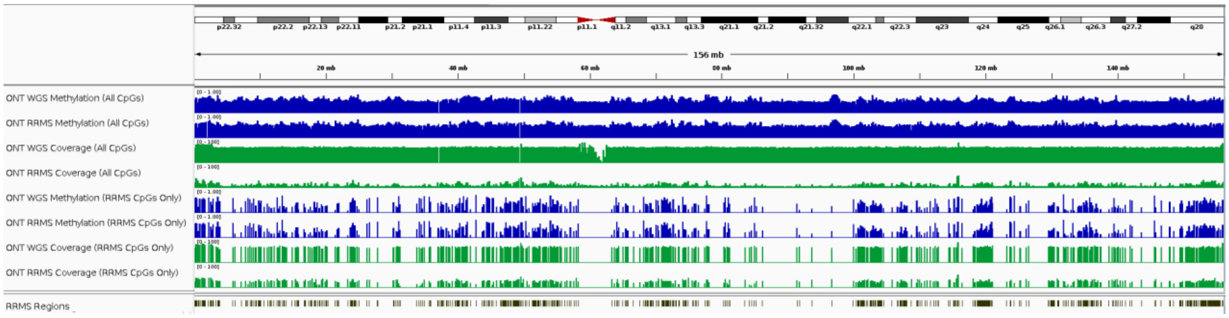

Chromosome Y

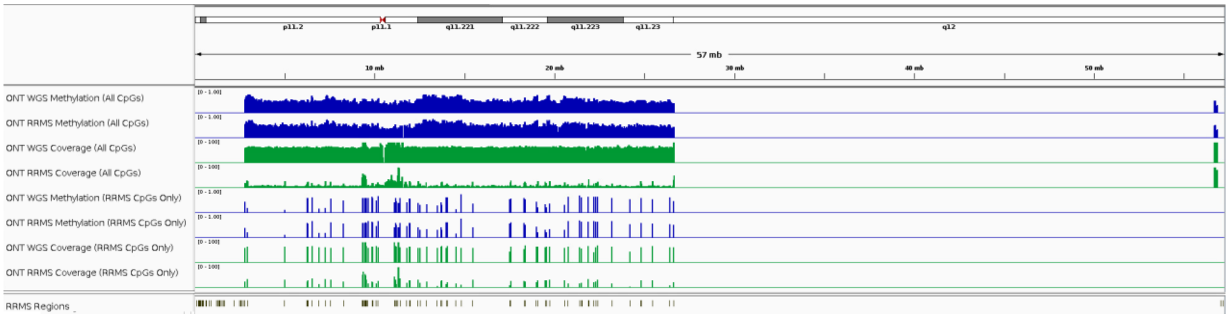

Supplement: Supplementary file 5 — Supplementary Data 6 [file 41467_2024_45778_MOESM5_ESM.pdf]
